# Genetic signatures of high-altitude adaptation and geographic distribution in Tibetan sheep

**DOI:** 10.1038/s41598-020-75428-4

**Published:** 2020-10-27

**Authors:** Jianbin Liu, Chao Yuan, Tingting Guo, Fan Wang, Yufeng Zeng, Xuezhi Ding, Zengkui Lu, Dingkao Renqing, Hao Zhang, Xilan Xu, Yaojing Yue, Xiaoping Sun, Chune Niu, Deqing Zhuoga, Bohui Yang

**Affiliations:** 1 Lanzhou Institute of Husbandry and Pharmaceutical Sciences of the Chinese Academy of Agricultural Sciences, Jiangouyan Street, Lanzhou, China; 2 Sheep Breeding Engineering Technology Research Center of Chinese Academy of Agricultural Sciences, Jiangouyan Street, Lanzhou, China; 3 China Agricultural Veterinarian Biology Science and Technology Co. Ltd, Xujiaping, Lanzhou, China; 4 Animal Husbandry Science and Research Institute of Gannan Tibet Autonomous Prefecture in China, Hezuo, China; 5 Pizhou Agricultural and Rural Bureau of Jiangsu Province, Parkway Street, Pizhou, China; 6 Pizhou Animal Health Supervision Institute of Jiangsu Province, Xizhong Street, Pizhou, China; 7 Institute of Livestock Research, Tibet Academy of Agriculture and Animal Science, Lhasa, 850000 China

**Keywords:** Gene expression profiling, Population genetics

## Abstract

Most sheep breeding programs designed for the tropics and sub-tropics have to take into account the impacts of environmental adaptive traits. However, the genetic mechanism regulating the multiple biological processes driving adaptive responses remains unclear. In this study, we applied a selective sweep analysis by combing 1% top values of *F*_*st*_ and *ZHp* on both altitude and geographic subpopulations (APS) in 636 indigenous Tibetan sheep breeds. Results show that 37 genes were identified within overlapped genomic regions regarding *F*_*st*_ significantly associated with APS. Out of the 37 genes, we found that 8, 3 and 6 genes at chromosomes (chr.) 13, 23 and 27, respectively, were identified in the genomic regions with 1% top values of *ZHp*. We further analyzed the INDEL variation of 6 genes at chr.27 (X chromosome) in APS together with corresponding orthologs of 6 genes in *Capra*, *Pantholops*, and *Bos Taurus*. We found that an INDEL was located within 5′UTR region of *HAG1* gene. This INDEL of *HAG1* was strongly associated with the variation of APS, which was further confirmed by qPCR. Sheep breeds carrying “C-INDEL” of *HAG1* have significantly greater body weight, shear amount, corpuscular hemoglobin and globulin levels, but lower body height, than those carrying “CA-INDEL” of *HAG1*. We concluded that “C-INDEL” variation of *HAG1* gene confers better hypoxia tolerance in the highlands of Tibetan and explains well geographic distributions in this population. These results contribute to our understanding of adaptive responses to altitude and geographic adaptation in Tibetan sheep populations and will help to guide future conservation programs for Tibetan sheep native to Qinghai-Tibetan Plateau.

## Introduction

Recent research data and model predictions indicate that increasing frequencies of abnormal weather events due to global climate change will have a fundamental impact on agricultural production^1^. For example, livestock experience numerous environmental stressors that have effects on both production traits, such as growth, reproductive performance, meat quality and adaptive traits such as cold tolerance^2,3^. In particular, the influence of climate change, i.e., cold stress on living organisms is exacerbated at the interface of extreme environments that typically occur in high altitude, plateaus and desert regions^4^. At such breeding programmes for livestock in these regions should include both adaptive and production traits^5^.

Tibetan sheep (*Ovis aries*) have the ability to adapt to a wide range of agro-ecological conditions, and represent an excellent model to gain new insights into genetic mechanisms underlying the adaptive response of livestock to extreme environments ^6,7^. This is also helpful to develop appropriate breeding programs under various scenarios of future climate change. Tibetan sheep have lived on the Tibetan plateau for thousands of years; however, the process and consequences of adaptation to this extreme environment conditions have not yet been elucidated ^8^.

Artificial selection during domestication and production-oriented breeding has greatly shaped the level of genomic variability in sheep. The genome of Tibetan sheep provides a unique opportunity for identifying signatures associated with selection. Array- or sequencing-based detection of the signatures of selection process has been described in cattle^9^, chicken^10^, dogs^11^, goat^12^, and in sheep^8,13^. Recently, the variation of many genes were identified to be strongly associated with as high-altitude adaptations in the Tibetan Mastiff (*EPAS1*)^8^, in yaks (*ADAM17*, *ARG2* and *MMP3*), Tibetan antelopes (*PKLR*, *ATP12A* and *NOS3*) and Tibetan wild boars (*ALB*, *GNG2* and *PIK3C2G*)^14–16^. However, each species may have different candidate genes responsible for high altitude adaptations, and investigations on selection signatures with respect to altitude and geographic adaptation were also less reported.

This study aimed to identify signatures evidence of recent selection among Tibetan sheep population for both altitude and geographic adaptation. We investigated selection signatures using whole-genomic variants in 15 indigenous Tibetan sheep populations. These populations consist of 636 sheep individuals living in the Qinghai-Tibetan Plateau areas in China. The overlapped genes underlying both altitude and environmental response were identified by screening genomic regions with significant values of both *ZHp* and *F*_*st*_ for autosome and hetersomes SNPs. We observed a small number of strong selection signatures near genes known to under strong artificial selection in other animals. Our findings can be used to better understand genomic signatures under selection controlling both adaptive traits in Tibetan sheep populations. The key genes identified in this study would be helpful to guide breeding practices for improvement of adaptive ability to extreme environments for sheep.

## Methods

### Ethics statement

All animals were handled according to the Guidelines for the Biological Studies Animal Care and Use Committee, People’ s Republic of China. Animal experiments were approved by the Animal Ethics Committee of the Institute of Animal Sciences of Chinese Academy of Agricultural Sciences.

### Classification of population from different adaptive traits

15 Chinese indigenous Tibetan sheep populations, consisting of 636 individuals derived from different altitudes ranging from 3000-5000 m were used. These indigenous Tibetan sheep populations living in the Qinghai-Tibetan Plateau areas in China were classified into different subpopulations to estimate the candidate genes responsible for altitude and geographic adaptation (Table 1). According to originated altitude information, two subpopulations were divided, i.e., lowland Tibetan sheep (< 3500 m): Guide Black Fur sheep (GD), Qilian White Tibetan sheep (QL), Tianjun White Tibetan sheep (TJ), Qinghai Oula Tibetan sheep (QH), Minxian Black Fur sheep (MX), Ganjia Tibetan sheep (GJ), Qiaoke Tibetan sheep (QK), and Gannan Oula Tibetan sheep (GN); highland Tibetan sheep (> 4500 m): Langkazi Tibetan sheep (LKZ), Jiangzi Tibetan sheep (JZ), Gangba Tibetan sheep (GB), Huoba Tibetan sheep (HB), Duoma Tibetan sheep (DM), Awang Tibetan sheep (AW), and Linzhou Tibetan sheep (LZ). For geographic regions, three different provinces in China were classified, Qinghai (QH, GD, QL, and TJ); Gansu (MX, GJ, QK, and GN); Tibetan (LKZ, JZ, GB, HB, DM, AW and LZ) (Table 1).

**Table 1:** Criterion of indigenous Tibetan sheep populations classified for different factors.

Types	Parameters	Subpopulation
Altitude	< 3500 m	GD, QL, TJ, QH, MX, GJ, QK, GN
	> 4500 m	LKZ, JZ, GB, HB, DM, AW, LZ
Geographic locations	Qinghai	GD, QL, TJ
	Gansu	QH, MX, GJ, QK, GN
	Tibetan	LKZ, JZ, GB, HB, DM, AW, LZ

### Physiological measurements of samples from different altitudes

To further elucidate the physiological significances of INDEL variation of *HAG1* gene based on the values of *F*_*st*_ and *ZHp*, we compared 4 out of 15 Tibetan subpopulations (GD, QL, AW and HB) due to the available phenotypic information. This selected subpopulations consist of 122 samples as listed in Table 2. Six parameters related to body size were determined: body weight/height/length, bust, chest depth, neck size, and two other traits, such as wool length, and shear amount (defined as the weight of the fleece post shearing)^17^; Thirteen physiological parameters: body temperature (Temperature), breathe rates (Breathe), pulse rates (Pulse), pulse interval (pulse interval), red-cell numbers (RBC), hematocrit, HCT, erythrocyte mean corpuscular volume (MCV)^17^, mean corpuscular hemoglobin (MCH), Mean corpuscular hemoglobin concentration (MCHC)^18^, Hemoglobin (HGB), Platelet (PLT), Red blood cell volume distribution width coefficient variation (RGW-CV), White blood cell count (WBC). Biochemical parameters: Glutamic pyruvic transaminase (ALT)^19^, Glutamic oxalacetic transaminase (AST) ^19^, Total protein (TP), Albumin (ALB), Globulin (GLO), Alkaline phosphatase (ALP), Lactate dehydrogenase (LDH), Cholinesterase (PCHE)^20^, Glucose (GLU), Total cholesterol (CHOL)^21^, Total calcium (CA); Nine blood-gas parameters: blood pH, pressure CO_2_ (pCO_2_), O_2_ saturation (O_2_S), concentration of HCO_3_^-^, standard bicarbonate, total CO_2_ concentration, Base excess (BE), Standard base excess (SBE); structural parameters related to Lung tissue: bronchial, thin bronchial, end thin bronchial, alveolar numbers per area (A_N_/m^2^), thick alveolar interval (TAI).

**Table 2:** Sampling information for the 15 indigenous Tibetan sheep populations used in this study.

Population	Population code	Sample number	Altitude (m)	Longitude and latitude	Sampling location
Guide Black Fur sheep	GD	39	3100	N:38°61′152″ E:103°32′160″	Senduo Town, Guinan County, Hainan Tibetan Autonomous State, Qinghai Province
Qilian White Tibetan sheep	QL	44	3540	N:42°20′178″ E:116°64′618″	Qilian Town, Qilian County, Delingha City, Mongolian Autonomous State, Qinghai Province
Tianjun White Tibetan sheep	TJ	64	3217	N:42°18′158″ E:116°42′210″	Shengge Countryside, Tianjun County, Delingha City, Mongolian Autonomous State, Qinghai Province
Qinghai Oula Tibetan sheep	QH	44	3630	N:34°16′433″ E:101°32′141″	Jianke Village, Kesheng Town, Henan Mongolian Autonomous County, Qinghai Province
Minxian Black Fur sheep	MX	67	3180	N:36°54′48″ E:103°94′107″	Taizi Village, Qingshui Town, Minxian County, Dingxi City, Gansu Province
Ganjia Tibetan sheep	GJ	58	3022	N:35°32′49″ E:102°40′802″	Xike Village, Ganjia Town, Xiahe County, Gannan Tibetan Autonomous State, Gansu Province
Qiaoke Tibetan sheep	QK	71	3410	N:35°42′106″ E:102°42′210″	Waeryi Village, Qihama Town, Maqu County, Gannan Tibetan Autonomous State, Gansu Province
Gannan Oula Tibetan sheep	GN	52	3616	N:33°51′312″ E:101°52′424″	Daerqing Administrative Village, Oula Town, Maqu County, Gannan Tibetan Autonomous State, Gansu Province
Langkazi Tibetan sheep	LKZ	10	4459	N:28°58′951″ E:090°23′757″	Kexi Village, Langkazi Town, Langkazi County, Shannan Territory of Tibet Autonomous Region
Jiangzi Tibetan sheep	JZ	46	4398	N:28°55′113″ E:089°47′692″	Reding Village, Cheren Town, Jiangzi County, Shannan Territory of Tibet Autonomous Region
Gangba Tibetan sheep	GB	85	4403	N:28°15′281″ E:088°24′787″	Yulie Village, Gangba Town, Gangba County, Rikaze Territory of Tibet Autonomous Region
Huoba Tibetan sheep	HB	34	4614	N:30°13′822″ E:083°00′249″	Rima Village, Huoba Town, Zhongba County, Rikaze Territory of Tibet Autonomous Region
Duoma Tibetan sheep	DM	8	4780	N:29°48′609″ E:091°36′191″	Sixth Village, Maqu Town, Anduo County, Naqu Territory of Tibet Autonomous Region
Awang Tibetan sheep	AW	5	4643	N:30°12′101″ E:098°63′098″	Ayi Third Village, Awang Town, Gongjue County, Changdou Territory of Tibet Autonomous Region
Linzhou Tibetan sheep	LZ	9	4292	N:29°09′121″ E:091°25′063″	Tanggu Village, Tanggu Town, Linzhou County, Tibet Autonomous Region

### Sample collection

We used 15 Chinese indigenous Tibetan sheep populations, consisting of 636 individuals, and four samples were included in each pool for each population. The detailed sampling information for the 15 indigenous Tibetan sheep populations, i.e., population code, sample number, altitude, longitude and latitude, sampling location, and geographic location, is shown in Table 2. The procedure of sampling liver and lung tissues was followed as mentioned previously^22^.

### DNA extraction and Sequencing

Briefly, for each Tibetan sheep, genomic DNA was extracted from liver and lung tissues using the QIAamp DNA blood mini kit (Qiagen, Germany) as described previously^23^. A260/280 ratio and agarose gel electrophoresis were used to evaluate the quality and integrity of the DNA. Genomic DNA was digested to a 300-400 bp fragment for library preparation, followed by end repair and ligated with an Illumina sequencing linker. The ligated products with sizes of 400-500 bp were loaded on 2% agarose gels and subsequently amplified via PCR. Libraries were sequenced by HiSeq 2500 sequencer (Illumina) in a 2 × 100 bp paired end mode. The sequencing coverage for each sample is averaged 10.3 × , within a range of 9–14 fold.

### Reads alignment and variant calling

Reads were aligned to the sheep reference genome ‘Ovis aries’ v4.0 (https://www.ncbi.nlm.nih.gov/assembly/GCF_000298735.2) using BWA (0.6.2-r126 version) followed by duplicate removal using Picard-Tools-1.55 (https://broadinstitute.github.io/picard/). The Genome Analysis Toolkit (GATK-2.6) was used to perform local realignment around existing INDELs and base quality score recalibration. Variant detection was performed using the GATK Unified Genotype Caller. To filter SNPs for downstream analysis, at least three reads with different mapping locations supporting the non-reference allele were present.

### Population genetics analysis

The pairwise genetic distance was determined relying on the number of allelic differences as described previously^23^. Briefly, the neighbor join tree was calculated based on the distance matrix using PHYLIP (version 3.69) ^24^. SNP pair with high correlation were removed via PLINK (PLINK, RRID: SCR 001,757) ^25^. The PCA and population structure were performed using EIGEN-SOFT (version 6.0.1) and FRAPPE software (version 1.1), respectively^26,27^. TreeMix was applied to estimate migration events, with migration numbers m = 0–5 as previously reported^28^. The selective sweep analysis was performed using VCFtools, v0.1.12b^29^, including θ (number of isolated sites), π (paired nucleotide difference), and Tajima's D. There are 500,000 SNPs randomly selected from the genome, and these SNPs were used to analyze the linkage disequilibrium *r*^2^ with Haploview^30^.

### Genome-wide Selective Sweep Test

To identify the functional genes underlying adaptive traits including altitude and geographic regions, we compared the overlapped genomic-regions by analyzing the signatures of selective effects on significant peaks. Two parameters relating to genome-wide selective sweep were used, i.e., *F*_*st*_ and *ZHp*. We calculated the genome-wide distribution of *F*_*st*_ values as previous reports^31^, for two subpopulations pairs as mentioned above, as to originated altitude information, and geographic regions (Table 2), using a sliding-window approach (100-kb windows with 50-kb increments). To identify regions that were likely to be or have been under selection, the “Z transformed heterozygosity” (*ZHp*) approach was used, as previously described ^32^. Individual *H*_*p*_ values were Z transformed as follows: *ZHp* = (*H*_*p*_-*μH*_*p*_)/*σH*_*p*_, where *μH*_*p*_ is the overall average heterozygosity and *σH*_*p*_ is the standard deviation for all windows within each population. We calculated the *ZHp* value in sliding 150-kb windows along the autosomes from sequence reads corresponding to the most and least frequently observed alleles at all SNP positions as previously described^33^.

### Bioinformatics analysis of population specific SNPs/INDELs

Overlapped candidate genes across altitudes and geographic regions were chosen for further analysis. In addition to this, we compared gene sequence of *Capra hircus*, *Pantholops hodgsonii*, and *Bos taurus* from NCBI database (https://www.ncbi.nlm.nih.gov/genome). We specifically focused on SNPs within genes and 1,000-bp upstream and downstream flanking regions of the genes (defined as the coordinates for the 3′ and 5′ UTR from ‘Ovis aries’ v4.0). Protein–protein interaction network was used to compare potential interactive genes with key overlapped genes in *Homo sapiens* from STRING database^34^.

### Validation of interactive genes

Expression of putative proteins interacted with key overlapped candidates related to genetic variation of originated altitudes and geographic regions were confirmed by qPCR. The liver and lung tissues from four euthanized individual sheep for each population were collected. Lower edge of either left liver or left lung were sampled, and immediately stored in liquid nitrogen. Total RNA was extracted from liver and lung samples with Trizol reagent (Invitrogen, Carlsbad, CA, USA). Complementary DNA (cDNA) from 1 μg of total RNA was synthesized using the ExScript RT reagent Kit (Takara Biomedicals, Tokyo, Japan). Detailed procedures for real-time PCR amplification were performed following standard protocol of SYBR Green real-time PCR kit (Applied Biosystems SYBR Green, MN, USA). The amplification reaction conditions were as follows: 95 °C for 3 min followed by 40 cycles of 95 °C for 10 s and 60 °C for 30 s. The primer is listed in Table S1. *GAPDH* gene was used as internal control, using $2^{{ - \Delta {\text{Ct}}}}$ method^35^. Four biological replicates and three technical replicates for each biological replicate were conducted.

## Results and discussion

### Genomic sequencing and PCA in 15 indigenous Tibetan sheep populations

In this study, we aimed to identify the candidate genes regulating multiple adaptive traits, including altitude and geographic regions. To accomplish this, we sequenced 15 indigenous Tibetan sheep populations consisting of 636 individuals, originated from different geographic locations (Table 2), via high-resolution whole genomic sequences techniques (WGS). To dissect the genomic heterozygosity levels and recombination events, we classified the populations into different altitude and geographic subpopulations (APS), including high altitude hypoxia (> 4500 m) vs. low altitude hypoxia (< 3500 m), and three different geographic locations, i.e., Qinghai, Gansu, and Tibetan (Table 1). WGS was performed on an Illumina HiSeq 2500 platform by using the pooled DNA from each population. Genome sequencing yielded an average of 40.84 Gb raw data, and produced 204 to 362 million sequence reads per population (Table S2). Over 90.75% of clean sequence reads were mapped to the newly annotated sheep reference genome (‘Ovis aries’ v4.0), indicating that high quality sequences were obtained (Table S3). Our efforts yielded an average sequence coverage of 10.3 × per sample, within a range of 9–14 fold. Single-nucleotide polymorphisms (SNPs) varied from 13–16 million for each population (Table S4).

More than 9 million SNPs for each Tibetan sheep population that confidently remained after filtering were used in the subsequent analyses. Results from SNPs statistics showed that 63% SNPs were identified at intergenic regions, whereas only 0.7% SNPs cases were found within exon regions (Table S5).

**Figure 1:**
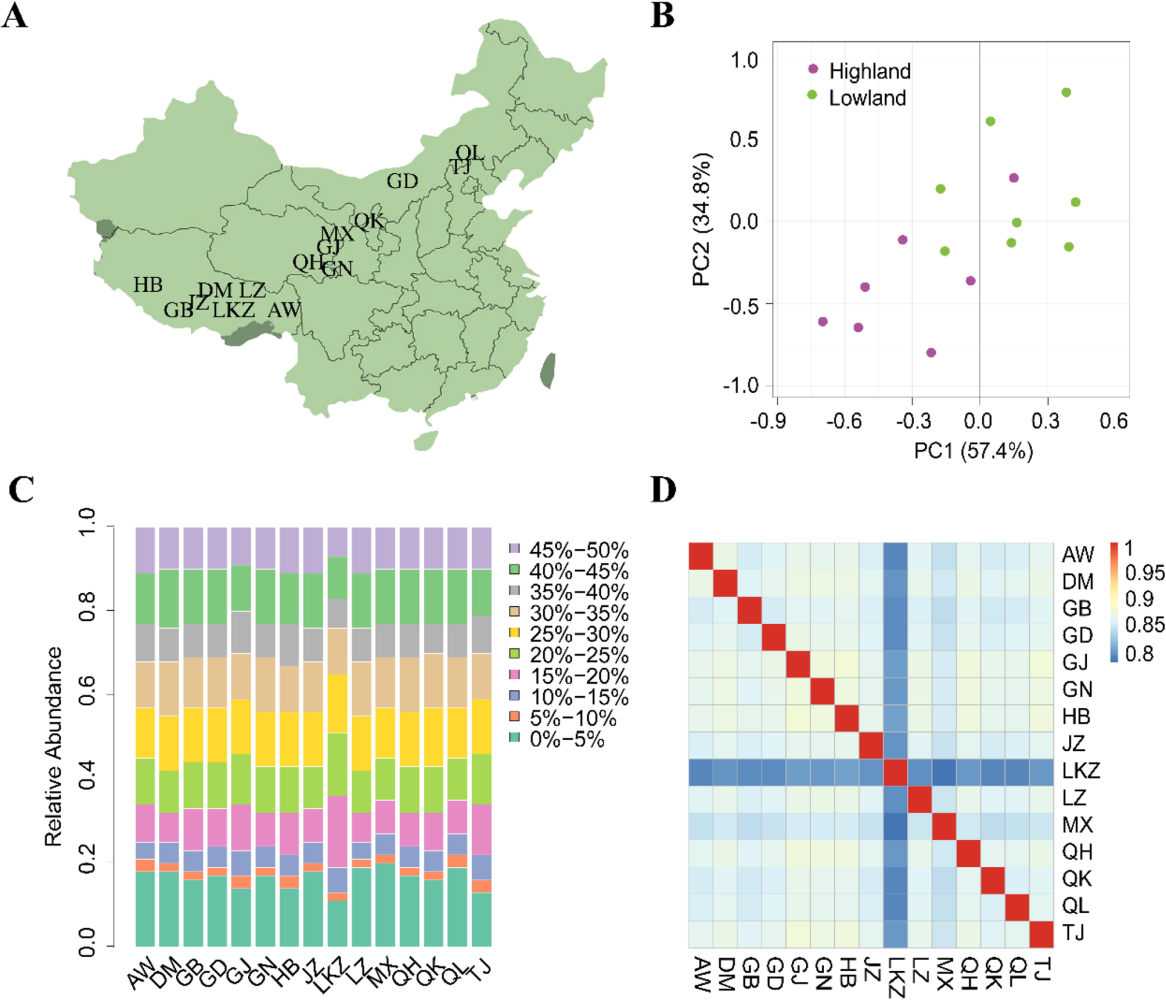
SNP analysis and population structure for 15 indigenous Tibetan sheep populations. A: distributive map of 15 indigenous Tibetan sheep populations living in the Qinghai-Tibetan Plateau areas in China used in this study; The map inserted in panel A was referred to the Fig. 2 from Deng et al.^53^ with slight modifications. B: Principal component analysis on SNP dataset after quality control in different Tibetan sheep populations; C: Distribution of minor allelic frequency (MAF) with 10 continued classes from 0–0.05 to 0.45–0.50; D: Relatedness of similarity index (IS) values in 15 Tibetan sheep populations.

Principal components analysis (PCA) was performed to examine the genetic separation of 15 indigenous Tibetan sheep populations originated from the Qinghai-Tibetan Plateau areas in China (Fig. 1A). PCA results from SNPs after quality control clearly showed that most Tibetan sheep populations have high genetic homogeneity, and cumulative explained variance for genetic differentiation in 15 Tibetan sheep populations from PC1 and PC2 was 92.2% (Fig. 1B). After calling SNP, we obtained ~ 9.3 million SNPs across the Tibetan sheep populations. The distribution of minor allelic frequency (MAF) with 10 continued classes from 0–0.05 to 0.45–0.50 for each population was observed (Fig. 1C). MAF of SNPs across 15 indigenous Tibetan sheep populations showed MAF within the range of 0–5% had most relative abundance with around 20% of total SNPs, whereas 5–15% MAF showed relatively low abundance with less than 3% across 15 indigenous Tibetan sheep populations. The distribution pattern of MAF among Tibetan sheep populations was similar except for LKZ (Fig. 1C). Similarity index (IS) value was used to estimate the homogeneity of SNPs across 15 indigenous Tibetan sheep populations. LKZ showed lowest similarity with other Tibetan sheep populations, which is consistent with MAF distribution results (Fig. 1D).

### Overlapped selective signals regarding genomic heterozygosity among altitude and geographic regions distributions

**Figure 2:**
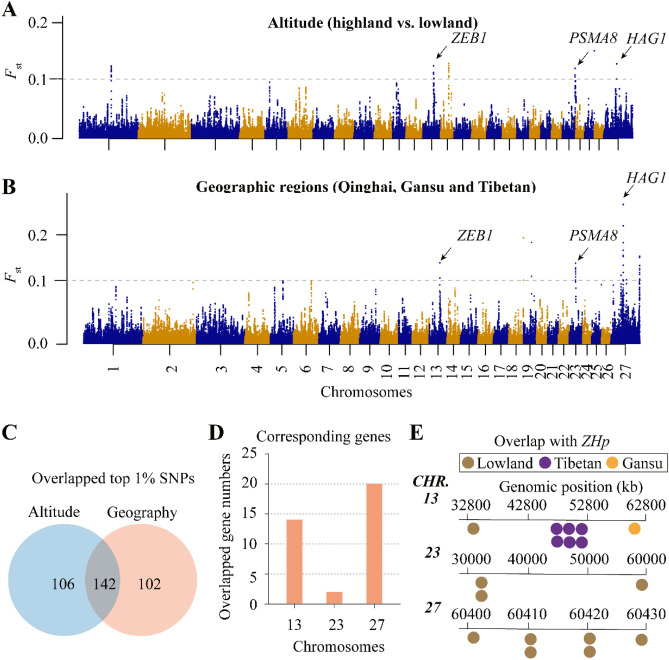
Manhattan analysis and candidate genes exploration. A-B: Manhattan plot representing *F*_*st*_ values for each SNP across Chromosomes for two subpopulations of altitude and geography. The genomic region with significant *F*_*st*_ values were highlighted in arrows. C: Venn diagram representing overlapped SNPs for two subpopulations of altitude and geography. D: Numbers of corresponding genes harboring overlapped SNPs for two subpopulations of altitude and geography. E: overlapped genes with 1% top values between *F*_*st*_ and *ZHp*.

*F*_*st*_ values were used to analyze the overlapped genes in the signatures of selection analysis on significant peaks for each of the different APS. It also named fixation index, typically used to evaluate differentiated genomic regions and identify selective signals among whole-genomic sequences^31,36^. *F*_*st*_ values over the whole genome based on genetic differentiation in APS were classified by altitude, and geographic locations (Fig. 2A-B). We found in total 248 and 244 SNPs with top 1% *F*_*st*_ values for APS, respectively (Fig. 2C). The numbers of overlapped candidate genes were presented based on top 1% *F*_*st*_ values for APS from 150-kb windows (Fig. 2D). There are three chromosomes (chr.) including chr.13, chr.23, and chr.27 (X chromosome), harboring significant SNPs, corresponding to 14, 3, and 20 genes, respectively (Fig. 2D). In addition, we compared these overlapped genomic regions with top 1% values for both *ZHp* and *F*_*st*_ to better understand genomic heterozygosity in specific populations. We found that *ZEB1* gene at chr.13 is identified with overlapping in lowland subpopulation with top 1% values for both *ZHp* and *F*_*st*_ (Table S6). It was reported that *ZEB1* is a target gene of hypoxia-inducible factors (*HIFs*), which is critical in the regulation of the macrovascular angiogenic response but not that of microvascular angiogenesis^37^. This indicates that *HIFs* can potentially be important transcription factors that involved in regulatory pathways of the available oxygen in the cellular environment under high-altitude conditions^37^. Moreover, it was reported that *HIF-1α* can stimulate the expression of *TWIST1* and *ZEB1* genes under hypoxic conditions^38,39^, suggesting potential functions of *ZEB1* gene in adaptive response of Tibetan sheep to hypoxic conditions.

We also identified six genes (*LOC101110166, PANK2, RNF24, VPS16, PCED1A* and *PTPRA*) with top 1% values for both *ZHp* and *F*_*st*_ in Tibetan geographic region of China (Fig. 2E; Table S6). Notably, one gene (*RALY*) is identified with overlapping with Gansu geographic region of China (Table S6). This gene (*RALY*) has been reported as a candidate gene involved in spliceosomal complex pathway in high-altitude adaption Tibetan sheep populations, indicating its important roles in hypoxia response in addition to *ZEB1*^13,37^. Moreover, we found three genes (*PSMA8*, *TAF4B*, *LOC105605498*) and six genes (*LOC101119700*, *LOC101119112*, *APOOL*, *ZNF711*, *LOC101123097* and *POF1B*) were identified in chr.23, and chr.27, respectively, with top 1% values for both *ZHp* and *F*_*st*_ in lowland subpopulation (Fig. 2E; Table S6).

### Haplotypes of HAG1 are related to adaptive response to environments and body size

**Figure 3:**
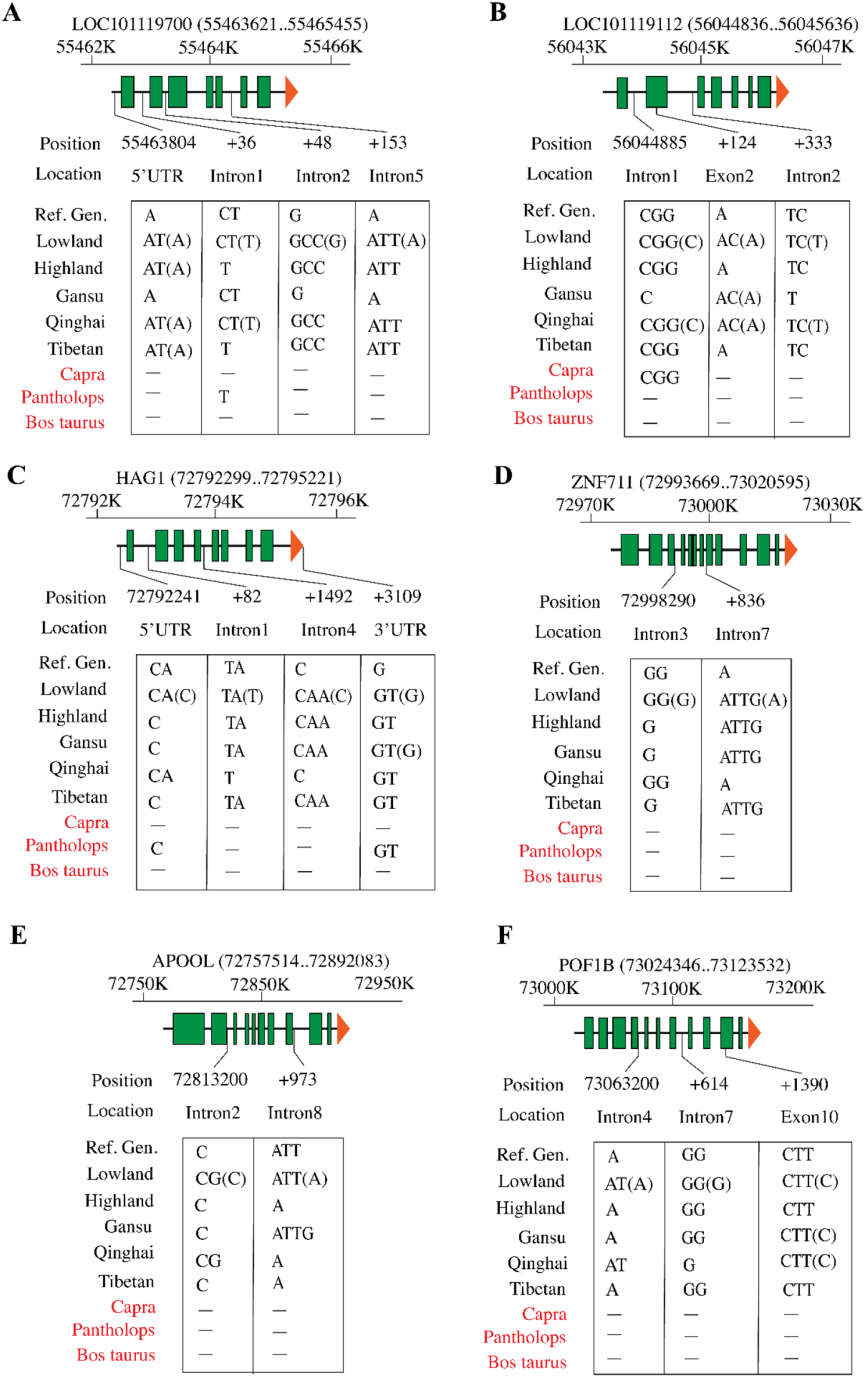
Gene INDEL analysis for 6 candidate overlapped genes at hetersomes within overlapped genomic regions by combing 1% top values of *F*_*st*_ and *ZHp*. A-F: INDEL analysis on 6 genes within clustered subpopulations in addition to three other species including *Capra*, *Pantholops*, and *Bos taurus*. The 6 genes were listed in Table S6.

Heteromorphic X chromosome, where one sex has two different types of sex chromosomes, face very different evolutionary consequences than do the autosomes. To further analyze the natural variations of these 6 overlapped genes on X chromosome (chr.27), we analyzed INDELs of these genes including 2 k-bp promoter and CDS regions together with other species, such as *Capra*, *Pantholops*, and *Bos Taurus* (Fig. 3). Notably, these species, such as *Bos Taurus* are considered as ideal models to uncover the mechanism of animal adaption to highland altitude hypoxia^16^. Results from INDEL information showed that 62% INDEL events were identified at intergenic regions, whereas only 0.3% INDEL cases were found within exon regions (Table S7). The INDEL happens in different types, such as deletion and insertion compared to reference genomes across the 6 overlapped genes (Fig. 3). Interestingly, our findings suggested that among 6 genes, the variation of INDEL at 5′-flank region of *HAG1* gene (*LOC101123097*) at 72,792,241 is in well line with the variations of APS (Fig. 3C); while the INDEL in other genes were either found in gene intron region or mixed INDELs types (Fig. 3A-F). INDEL of “C” on 5′UTR of *HAG1* gene was observed in Tibetan sheep from highland, and Tibetan geographic region, we named here as “C-INDEL”, while INDEL of “CA” is occurred in Qinghai, we hence named as “CA-INDEL”. Interestingly, we found there are two INDEL types mixed in lowland altitude (Fig. 3C).

**Figure 4:**
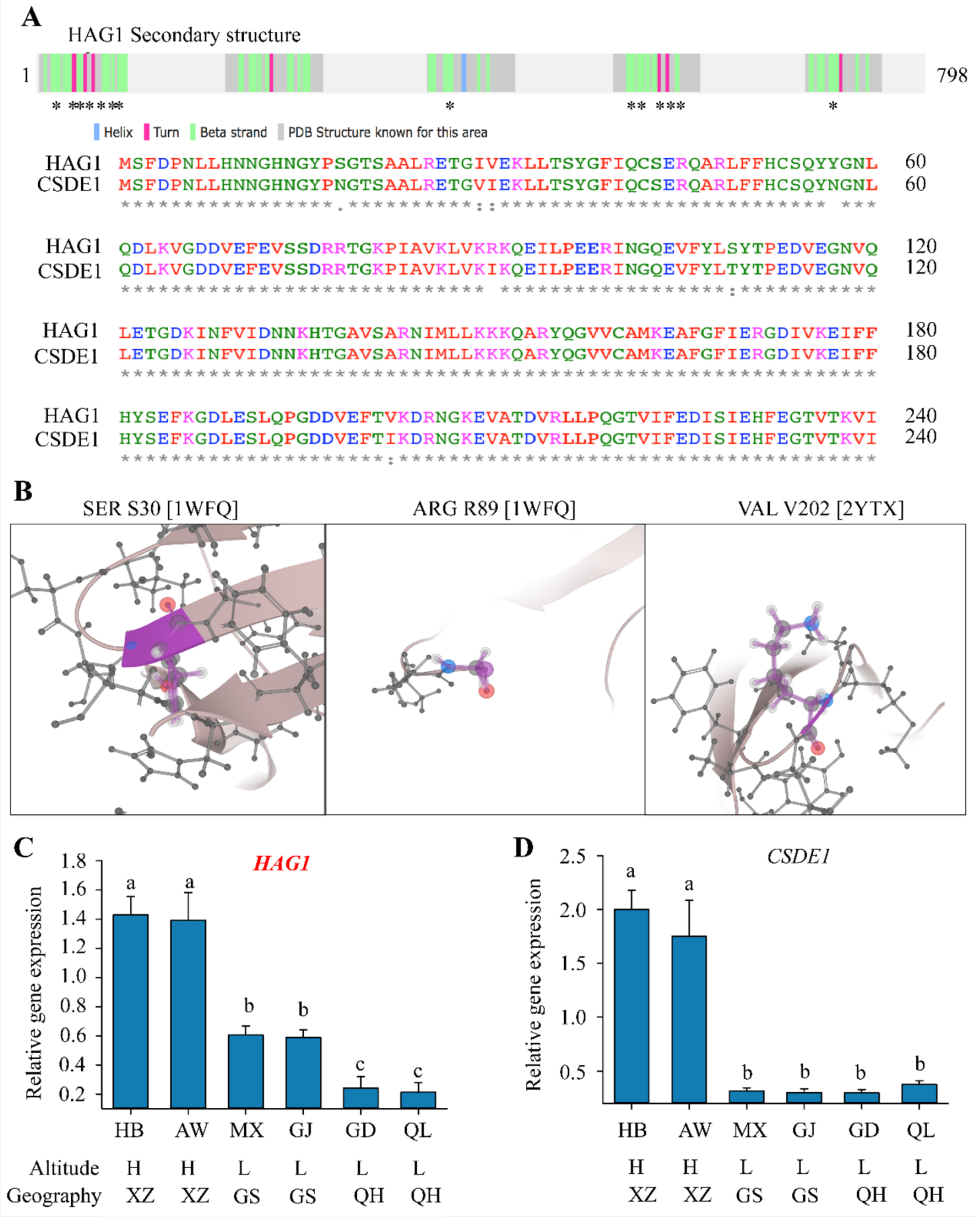
Comparison on sequence alignments and expression pattern between *HAG1* and *CSDE1*. A: Secondary structure of *HAG1* and differentiated amino acids between *HAG1* and *CSDE1*. The location of 7 differentiated amino acids were depicted in symbol “*”, which are majorly on beta-strand and turn. Secondary structure of protein is predicted via Uniprot database from different PDB entries. B: Differentiated location of amino acids on representative 3D protein structure; C-D: Comparison on expression levels of *CSDE1* and *HAG1* in given 6 indigenous Tibetan sheep populations in this study. The 6 indigenous Tibetan sheep populations include Guide Black Fur sheep (GD), Qilian White Tibetan sheep (QL), Minxian Black Fur sheep (MX), Ganjia Tibetan sheep (GJ), Huoba Tibetan sheep (HB) and Awang Tibetan sheep (AW). Expression levels were calculated against GAPDH using mRNA from live and lung tissues, and four biological replicates were conducted.

We further analyzed the protein structure and gene expression variation of *HAG1* among different indigenous Tibetan sheep (Fig. 4). According to bioinformatics analysis, we predicted the protein sequences of *HAG1* using protein translation tool from ExPASy database (https://web.expasy.org/translate/), and found the similarities score reached up to 99% in either mRNA or protein sequences between *CSDE1* and *HAG1* protein, indicating both *HAG1* with *CSDE1* are likely to be involved in similar regulatory pathway. Notably, the *CSDE1* (Cold Shock Domain Containing E1), alternative name: *UNR*, is a conserved RBP containing five cold-shock domains (CSDs) that bind single-stranded RNA^40,41^. Function of proteins possessing CSDs are involved in two processes: transcriptional and translational control. Two major cold shock proteins CspA and CspB in *Escherichia coli* and *Bacillus subtilis*, respectively, were intensively reported^40^. CspA and CspB are massively and transiently induced after a temperature downshift and are involved in the adaptation to cold shock^42^.

*HAG1* protein consists of 798 amino acids. We then compared the secondary structure of *HAG1* with known *CSDE1*, and found most identified residues are conserved, the similarity between *HAG1* and *CSDE1* proteins were 87% from NCBI protein blast website (blast.ncbi.nlm.nih.gov/). Most differential regions were observed at N-terminal of protein containing 7 different amino acids, and these amino acids were located in either Beta strand or Turn domains, but not Helix (Fig. 4A). These domains were reported to represent the most evolutionarily conserved nucleic acid-binding protein domain, as found in bacteria and eukaryotes^43,44^. Interestingly, this kind of Beta strand domain can also mediate the binding affinity to single-stranded DNA and RNA^45,46^. Three different amino acids between *HAG1* and *CSDE1*, i.e., S30, R89 and V202, were highlighted and mapped at 3D protein structure from two PDB entries (1WFQ and 2YTX) according to Uniprot database (CSDE1_Human Protein ID: 075,534) (Fig. 4B).

The 5′ untranslated region (5′-UTRs) are known to regulate gene expression. INDEL between different subpopulations at the region of 5′-UTRs is expected to influence different expression of *HAG1* gene. To compare the expression levels between *HAG1* and *CSDE1*, we used 6 indigenous Tibetan sheep populations, including Guide Black Fur sheep (GD), Qilian White Tibetan sheep (QL), Minxian Black Fur sheep (MX), GanjiaTibetan sheep (GJ), Huoba Tibetan sheep (HB) and Awang Tibetan sheep (AW). We sampled from liver and lung tissues for qPCR as same position for DNA sequencing. Notably, it was reported that *CSDE1* gene is high expressed in muscle and fetal ovary according to sheep gene expression atlas dataset (https://biogps.org/sheepatlas/) and as documented elsewhere^47^. We also found that *HAG1* gene from liver and lung tissues is highly expressed in Tibetan sheep from highland regions, rather than that from lowland regions (Fig. 4C). *HAG1* gene also showed distinct expression pattern among different geographic locations (Fig. 4C). Interestingly, *CSDE1* gene exhibited clear differences, measured in liver and lung tissues for the Tibetan sheep between highland and lowland, but not for the sheep from different geographic regions (Fig. 4D).

### Function of *HAG1* in adaptive characteristics

Therefore, an interesting question arising here is whether interactive proteins with *CSDE1* could show similar expression pattern between the subpopulations of altitude and geographic region? Therefore, we reconstructed the *CSDE1*-centerized module according to STRING protein-interaction database (Figure S3A-B; Table S8). Differential gene expression analysis via qPCR suggested that 9 genes in the module have strong correlation with *CSDE1* and *HAG1* (Figure S3B; Table S8). These are: *MAX, MYC, YBX1, HNRNPU, DHX9, PABPC1, PAIP1, STRAP,* and *SYNCRIP*. Previous reports showed that the mRNA of *CSDE1* is up-regulated in a high percentage of skin and ovary cancers, and *UNR*/*CSDE1* regulates critical Melanoma genes, including *PABPC1* gene^48^. Besides, *CSDE1* regulates internal ribosome entry site (IRES)-dependent translation of the transcripts encoding the oncogene *MYC*^49^. Notably, *PABPC1* and *MYC* show significantly distinct difference between highland and lowland subpopulations as *CSDE1* (*p* < 0.05), but do not show clear difference in different geographic distribution as *HAG1* (Figure S3C-D), although expression levels of *HAG1* exerted strongly positive correlation with that of *CSDE1* as well as its 9 interactive genes (Figure S3B; Table S8). These evidences reveal that the function of *HAG1* gene might be also involved in other biological process.

### Morphological and physiological parameters of different haplotypes

**Figure 5:**
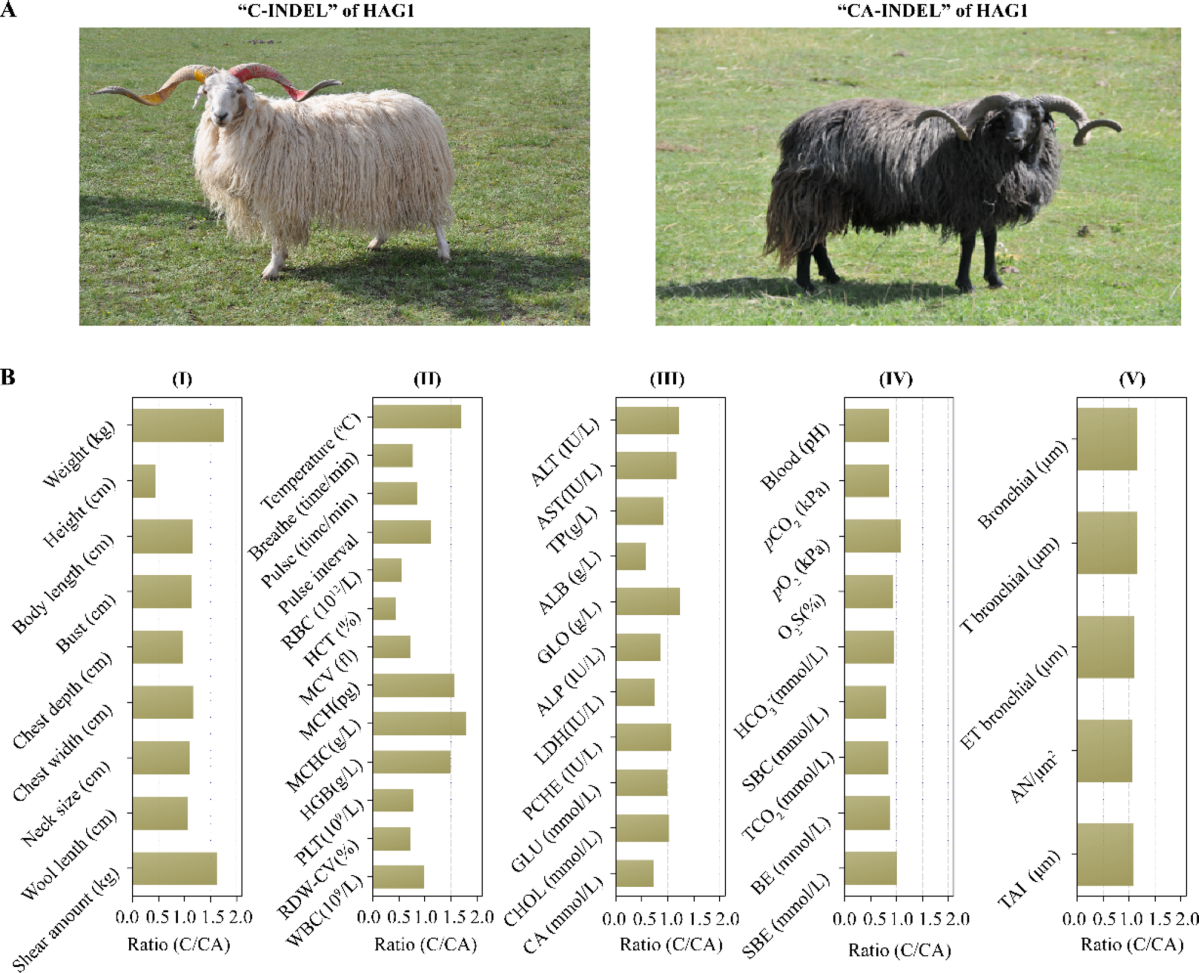
Comparison on morphological and physiological traits in 15 Tibetan sheep populations from 4 representative Tibetan sheep subpopulations (GD, QL, AW and HB), consisting of 122 samples with different INDEL of *HAG1* gene. A: Representative picture of indigenous Tibetan sheep (Huoba Tibetan sheep, HB for “C-INDEL”, and Guide Black Fur sheep, GD for “CA-INDEL” originated between two haplotypes promoter of *HAG1*; B: Comparison on different physiological parameters of 15 Tibetan sheep between “CA-INDEL” and “C-INDEL” of *HAG1*. The first types include GD and QL, while the latter types include AW and HB. The averaged values for each parameter were derived from at least 10 biological replicates.

Finally, to interpret the biological functions of *HAG1*, we selected 4 indigenous Tibetan sheep populations containing 15 individuals in total with contrasting expression pattern of *HAG1*. AW and HB belonging to “C-INDEL” show high expression levels of *HAG1*, as presented in Fig. 5A, while GD and QL belonging to “CA-INDEL” show low expression levels from liver and lung tissues (Fig. 5B). We then compared the ratio of “C-INDEL” over “CA-INDEL” in 47 physiological and biochemical parameters (Fig. 5B). Results show two Tibetan sheep breeds possessing “C-INDEL” of *HAG1* have significantly greater body weight, shear amount, chest width and body length, but have lower body height, relative to sheep in subpopulations with “CA-INDEL” of *HAG1* (*P* < 0.005) (Fig. 5B).

Our study showed that certain haplotype of *HAG1* could be an indicator for both adaptive response and production traits, such as altitude hypoxia (adaptive response), body weight (production traits), shear amount (production traits), and body length (production traits) (Fig. 5B). This is consistent with the conclusion that large physical size in highland as observed in human^50^. In particular, body weight of Tibetan sheep in “C-INDEL” population is 1.7 times higher than that in “CA-INDEL” group. For body haematological parameters, body temperature, pulse interval, MCH, MCHC, HGB levels are at least 1.5 times higher in Tibetan sheep population with “C-INDEL” of *HAG1* than that with “CA-INDEL”; this trend is similar to that observed in Tibetan sheep and dogs from different altitudes^13,51^. Interestingly, breathe rates, pulse rates, RBC, HCT, MCV, PLT, and RDW-CV are relatively lower in “C-INDEL” of *HAG1* than that in “CA-INDEL” Tibetan sheep population. Values of blood-gas parameters were similar or relatively lower in “C-INDEL” population than that in “CA-INDEL” population, except for pCO_2_. For lung tissue structure parameters, values are relatively higher in “C-INDEL” population than that in “CA-INDEL” population. In particular, elevated lactate dehydrogenase (LDH) in “C-INDEL” population relative to “CA-INDEL” population support the findings in other sheep studies on hypoxemia response^52^.

## Conclusion

This study presents a strategy to uncover the potential genes that underlie both altitude and geographic adaption through selective sweep analysis on genomic sequence of 15 indigenous Tibetan sheep populations. INDEL “C” within 5′-UTR of *HAG1* gene was strongly associated with higher expression levels of *HAG1* gene and better adaptive ability to high altitude, than that in those INDEL “CA” Tibetan sheep breeds. We concluded that the elite INDEL of *HAG1* gene could potentially enhance Tibetan sheep breeding programmes.

## Supplementary information

Supplementary Information 1.

## Abbreviations

HAG1: Heterosome altitude and geographic gene
Temperature: Body temperature
Breathe: Breathe rates
Pulse: Pulse rates
pulse interval: Pulse interval
RBC: Red-cell numbers
HCT: Hematocrit
MCV: Erythrocyte mean corpuscular volume
MCH: Mean corpuscular hemoglobin
MCHC: Mean corpuscular hemoglobin concentration
HGB: Hemoglobin
PLT: Platelet
RGW-CV: Red blood cell volume distribution width coefficient variation
WBC: White blood cell count
ALT: Glutamic pyruvic transaminase
AST: Glutamic oxalacetic transaminase
TP: Total protein
ALB: Albumin
GLO: Globulin
ALP: alkaline phosphatase
LDH: Lactate dehydrogenase
PCHE: Cholinesterase
GLU: Glucose
CHOL: Total cholesterol
CA: Total calcium
pCO_2_: Pressure CO_2_
O_2_S: O_2_ saturation
BE: Base Excess
SBE: Standard Base Excess
A_N_/m^2^: End thin bronchial, alveolar numbers per area
TAI: Thick alveolar interval
GD: Guide Black Fur sheep
QL: Qilian White Tibetan sheep
TJ: Tianjun White Tibetan sheep
QH: Qinghai Oula Tibetan sheep
MX: Minxian Black Fur sheep
GJ: Ganjia Tibetan sheep
QK: Qiaoke Tibetan sheep
GN: Gannan Oula Tibetan sheep
LKZ: Langkazi Tibetan sheep
JZ: Jiangzi Tibetan sheep
GB: Gangba Tibetan sheep
HB: Huoba Tibetan sheep
DM: Duoma Tibetan sheep
AW: Awang Tibetan sheep
LZ: Linzhou Tibetan sheep
MAF: Minor allelic frequency
HIFs: Hypoxia-inducible factors
*CSDE1*: Cold Shock Domain Containing E1
CSDs: Cold-shock domains
