# Supplementary material for: Genetic signatures of high-altitude adaptation and geographic distribution in Tibetan sheep
Source: Sci Rep. 2020 Oct 27;10:18332. doi: 10.1038/s41598-020-75428-4 (PMC7591910; doi:10.1038/s41598-020-75428-4)
Supplement: Supplementary file 1 — Supplementary Information 1. [file 41598_2020_75428_MOESM1_ESM.docx]

**SUPPLEMENTARY INFORMATION**

**Genetic signatures of high-altitude adaptation and geographic distribution in Tibetan sheep**

Liu Jianbin^1,2＆^, Yuan Chao^1,2^, Guo Tingting^1,2^, Wang Fan^3^, Zeng Yufeng^1^, Ding Xuezhi^1^, Lu Zengkui^1,2^, Ding Kaorenqing^4^, Zhang Hao^5^, Xu Xilan^6^, Yue Yaojing^1,2^, Sun Xiaoping^1,2^, Niu Chune^1,2^, Deqing Zhuoga^7＆^,Yang Bohui^1,2＆^

Numbers of Supplemental Figures: 3

Numbers of Supplemental Tables: 8


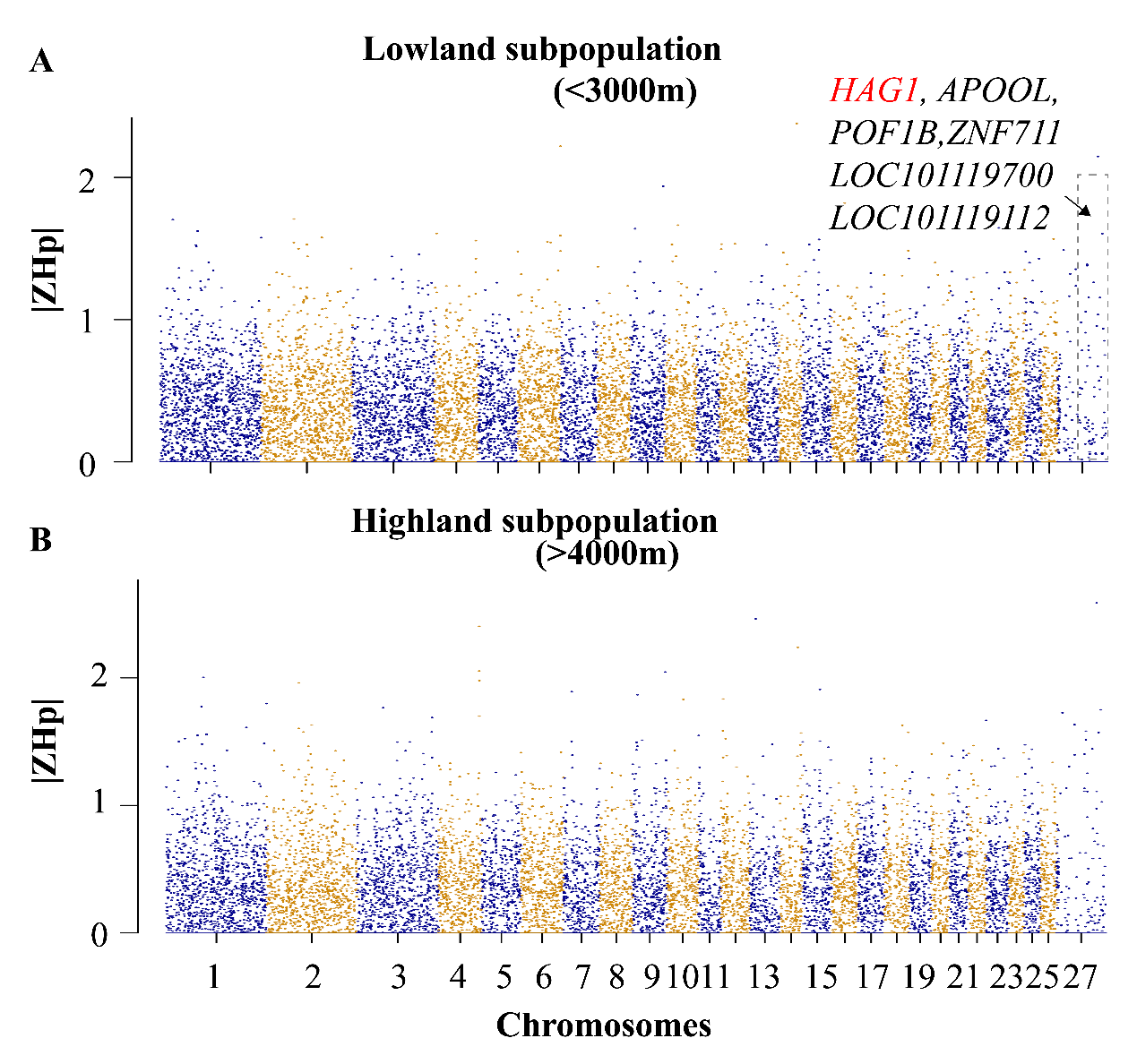


**Figure S1.** Manhattan plots representing the *|ZHp|* values between two altitude subpopulation. *|ZHp|* values in lowland subpopulation (A) and highland subpopulation (B). Names of genes overlapped with 1% top *F*_st_ values in hetersome (Chromosome 27) shown in frame were labeled in italic.


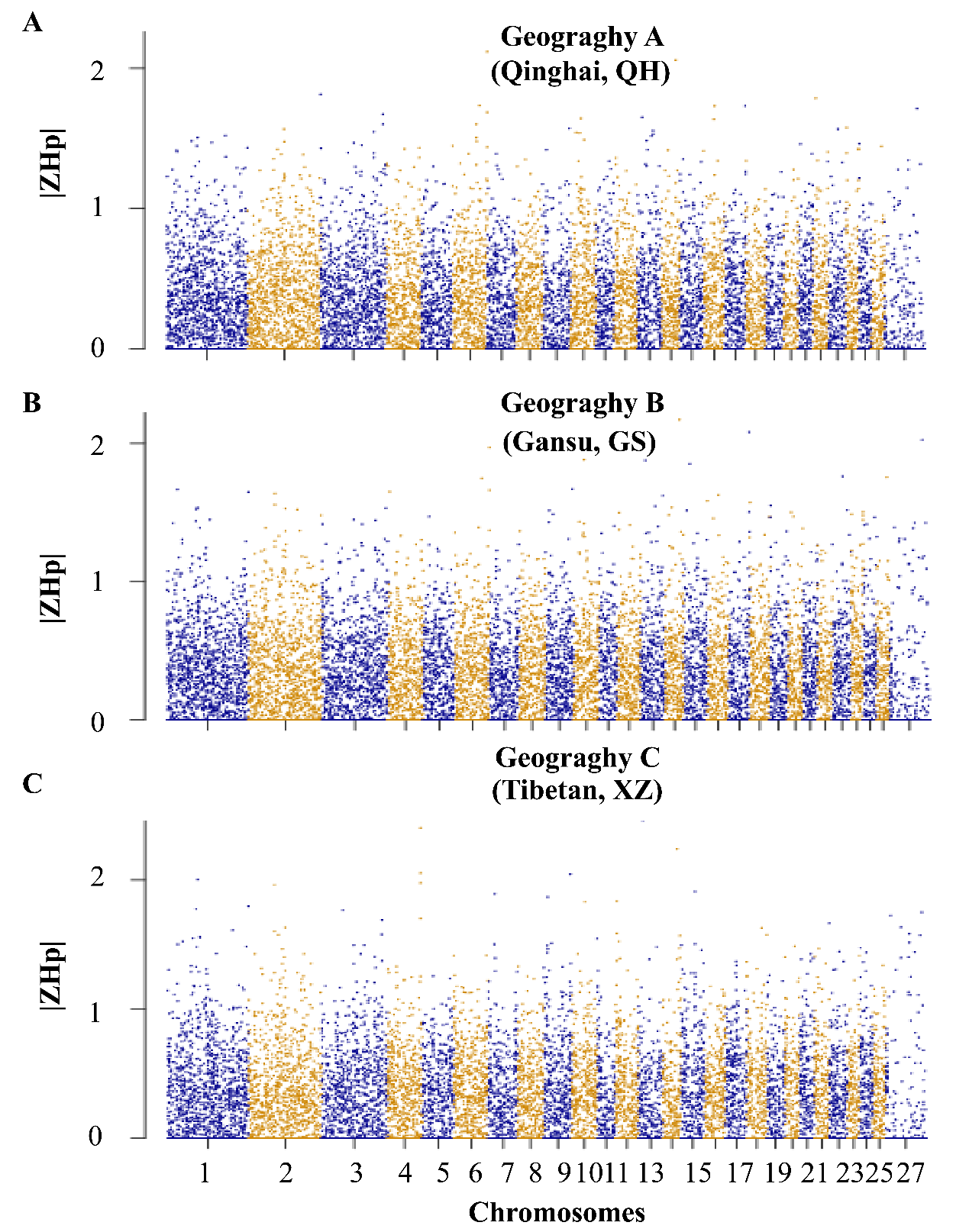


**Figure S2.** Manhattan plots representing the *|ZHp|* values of geographic regions of China. A-C: environmental adaptive response of genomic heterozygosity as indicated by *|ZHp|* values in specific Qinghai, Gansu, and Tibetan regions of China, respectively.


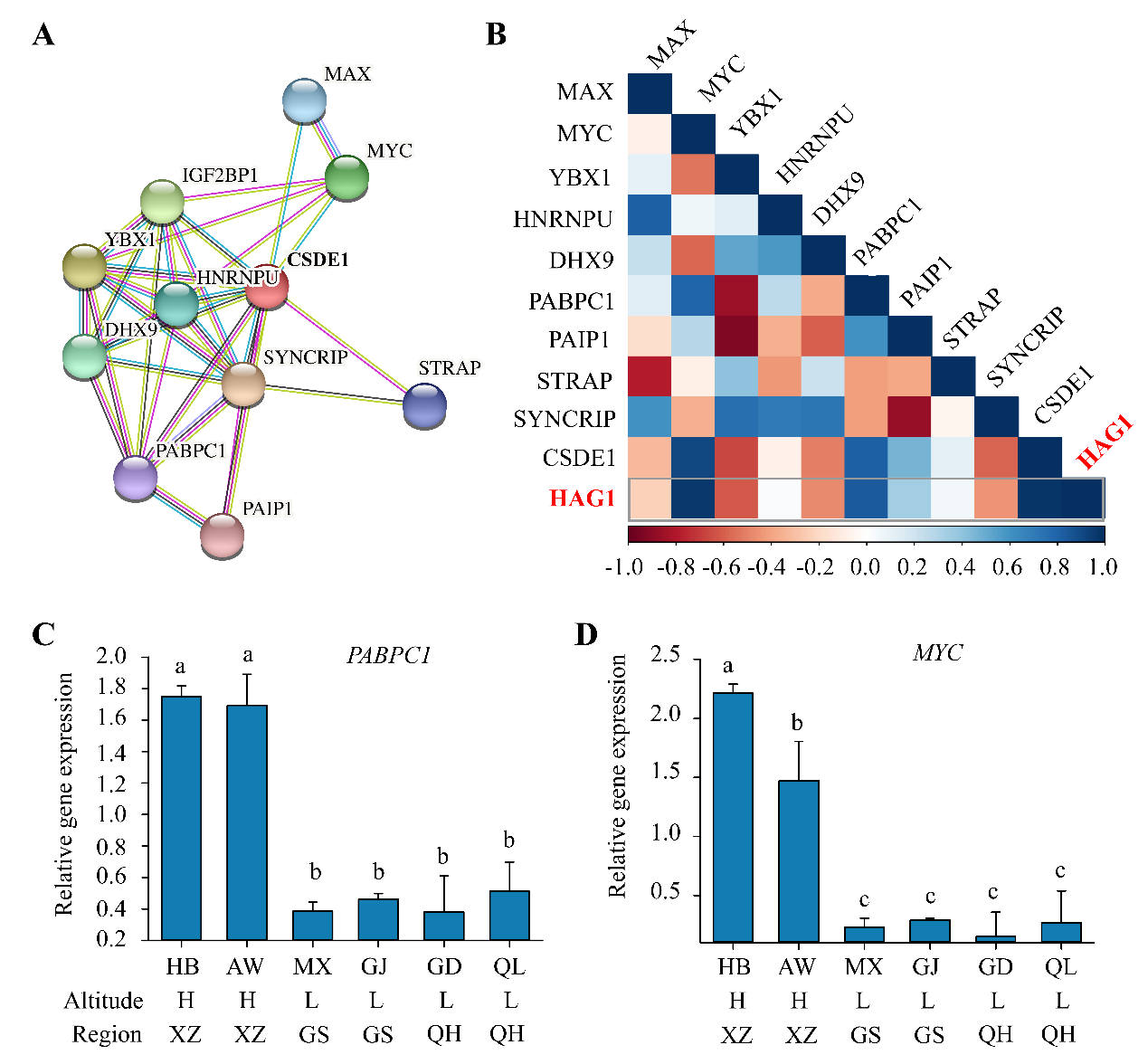


**Figure S3.** Gene expression correlation of *CSDE1* and its interactive proteins. A: *CSDE1*-interative protein network. The network was constructed by STRING database. There are 9 genes involved in the network. B: Relatedness of expression levels of 9 genes together with *CSDE1* in given 6 indigenous Tibetan sheep populations with different clusters. C-D: Comparison on 3 genes with strong correlation with *CSDE1*/*HAG1* in given 6 indigenous Tibetan sheep populations with different clusters. Pearson correlation coefficient, *r* across 3 genes (*MYC, PABPC1*, and *CSDE1*) with *CSDE1*/*HAG1* were more than 0.86. See abbreviation for 6 indigenous Tibetan sheep populations in Figure 4 and full names of sheep are shown in Table 1.

**Supplementary Table S1.** List of primer used in this study.

| **primer for CRISPR lines** | **Sequence** | | **Product size** | **Purpose** |
| --- | --- | --- | --- | --- |
| KLF4 | F | GTTAACTTTGCCACCGGGTT | 160 | Candidate gene screen |
|  | R | GTCCACATGGTGTGCGTTTA |  |  |
| PMSA1 | F | ACAAGGATCTCTGGCAGCAT | 139 | Candidate gene screen |
|  | R | TAACAGGCTTGGCTCCATCA |  |  |
| PAT1 | F | GGTGCGGAGTTGTGTTTGAT | 116 | Candidate gene screen |
|  | R | CAGTATGAGGCCTGTGTTGC |  |  |
| ZNF711 | F | GAGAGTGAGCGAGGAGAACA | 135 | Candidate gene screen |
|  | R | ATGTACTCCCGCAAGGTGAA |  |  |
| MAX | F | CTGTCACCTCTGAGCCTTCA | 100 | Candidate gene screen |
|  | R | GGCCTCACAATGTTGGTTGT |  |  |
| MYC | F | GTGCTGGTGCCTTAACTTCC | 184 | Candidate gene screen |
|  | R | CGCCTGAAATGCCGAGATAG |  |  |
| IGF2BP1 | F | CCAGTGCTACGACAGGTACA | 183 | Candidate gene screen |
|  | R | GAGAGGAAGAAGTCGCCGTA |  |  |
| YBX1 | F | TGGGCCATCACTGACATCTT | 125 | Candidate gene screen |
|  | R | ACCTCGGAGTAGCTATTGGC |  |  |
| DHX9 | F | TCACTTCTGGGCATGGTGAT | 124 | Candidate gene screen |
|  | R | ATGTCCGTCTTGCTTGCATC |  |  |
| HNRNPU | F | AGCTCCTGGTTGAAGAGCAT | 132 | Candidate gene screen |
|  | R | GAGTCCCGCATCCAATTTCC |  |  |
| SYNCRIP | F | GCAGACCTGCTAGCCATAGA | 93 | Candidate gene screen |
|  | R | CATGGCTCCATAACCCTCCA |  |  |
| STRAP | F | ATCCGACGTTCCTCTCTGAC | 86 | Candidate gene screen |
|  | R | GATCTTGCCTGCTGCCTTAC |  |  |
| PABPC1 | F | CTAAGCAGGCGAGCATCTTC | 85 | Candidate gene screen |
|  | R | GCCTTGATGAAACGGGACTC |  |  |
| PAIP1 | F | ACCTCGAGAGAGACCTGGAT | 245 | Candidate gene screen |
|  | R | CCAAGGAAGGTGCTCGACAA |  |  |
| ACTIN | F | GGTTCTTGGAGGGTCATGGG | 174 | Candidate gene screen |
|  | R | CACCCAATCACAGGAGGTGG |  |  |

**Supplementary Table S2.** Number of reads and bases in quality control

| **Raw reads** | **Raw bases** | **Filtered reads** | **Filtered Bases** | **Clean_Reads_Percent（﹪）** | **q20（﹪）** | **GC（﹪）** |
| --- | --- | --- | --- | --- | --- | --- |
| 348393806 | 49717966100（49.718G） | 313566970 | 44689987438（44.690G） | 90.00 | 89.89 | 44.00 |
| 226228158 | 33934223700（33.934G） | 206870306 | 30995692501（30.995G） | 91.44 | 91.34 | 42.00 |
| 240822344 | 36123351600（36.123G） | 219536642 | 32893946799（32.893G） | 91.16 | 91.06 | 42.00 |
| 243273026 | 36490953900（36.490G） | 222851860 | 33391842777（33.391G） | 91.60 | 91.50 | 42.00 |
| 395036688 | 55643391632（55.643G） | 361884692 | 50876175467（50.876G） | 91.61 | 91.43 | 43.00 |
| 235351580 | 35302737000（35.302G） | 214971604 | 32208661162（32.208G） | 91.34 | 91.23 | 42.00 |
| 376180596 | 52708575224（52.709G） | 345064778 | 48193881125（48.194G） | 91.73 | 91.43 | 43.00 |
| 239897984 | 35984697600（35.984G） | 217971432 | 32660034117（32.66G） | 90.86 | 90.76 | 42.00 |
| 267404132 | 40110619800（40.110G） | 238839078 | 35782055205（35.782G） | 89.31 | 89.20 | 41.50 |
| 238774548 | 35816182200（35.816G） | 214904894 | 32196373420（32.196G） | 90.00 | 89.89 | 42.00 |
| 242782222 | 36417333300（36.417G） | 218702292 | 32766117102（32.766G） | 90.08 | 89.97 | 43.00 |
| 252024238 | 37803635700（37.803G） | 229151150 | 34331141769（34.331G） | 90.92 | 90.81 | 43.50 |
| 233665026 | 35049753900（35.049G） | 204288278 | 30602068856（30.602G） | 87.42 | 87.31 | 42.00 |
| 242486420 | 36372963000（36.372G） | 223127380 | 33432380097（33.432G） | 92.01 | 91.91 | 42.00 |
| 393170830 | 55090898564（55.091G） | 360646508 | 50420217885（50.420G） | 91.73 | 91.52 | 43.00 |

**Supplementary Table S3.** Read mapping statistics and coverage of depth

| **Population** | **Samples** | **Mapped reads^a^** | **Unmapped reads** | **Mapping rate^b^** | **Average depth** | **Coverage at least 1X（﹪）** | **Coverage at least 10X（﹪）** |
| --- | --- | --- | --- | --- | --- | --- | --- |
| AW | 5 | 311072521 | 2494449 | 99.20 | 16.82 | 97.24 | 80.91 |
| DM | 8 | 206322028 | 548278 | 99.73 | 11.73 | 97.01 | 49.60 |
| GB | 85 | 218805345 | 731297 | 99.67 | 12.43 | 97.05 | 56.00 |
| GD | 39 | 222031918 | 819942 | 99.63 | 12.61 | 97.05 | 56.35 |
| GJ | 58 | 360317423 | 1567269 | 99.57 | 19.22 | 97.30 | 87.24 |
| GN | 52 | 214292303 | 679301 | 99.68 | 12.18 | 97.04 | 54.37 |
| HB | 34 | 342741513 | 2323265 | 99.33 | 18.16 | 97.29 | 84.78 |
| JZ | 46 | 217214134 | 757298 | 99.65 | 12.34 | 97.06 | 54.96 |
| LKZ | 10 | 237687305 | 1151773 | 99.52 | 13.49 | 97.11 | 63.97 |
| LZ | 9 | 214369139 | 535755 | 99.75 | 12.18 | 97.02 | 53.59 |
| MX | 67 | 217021463 | 1680829 | 99.23 | 12.33 | 97.03 | 53.25 |
| QH | 44 | 228366470 | 784680 | 99.66 | 12.96 | 97.05 | 56.47 |
| QK | 71 | 203433100 | 855178 | 99.58 | 11.55 | 97.01 | 47.74 |
| QL | 44 | 221872958 | 1254422 | 99.44 | 12.59 | 97.01 | 56.39 |
| TJ | 64 | 358874519 | 1771989 | 99.51 | 19.04 | 97.31 | 87.58 |

^a^ Mapped reads was calculated after PCR duplicates removed, one read could be mapped to multiple positions.

^b^ Mapping rate=Mapped reads/(Mapped reads+Unmapped reads)

**Supplementary Table S4.** Statistics for the 99 sequenced domestic sheep from 15 indigenous Tibetan sheep populations.

| **Population** | **Samples** | **Average genome coverage (%)** | **SNPs+Indels** | **SNPs** | **Coding SNPs** | **Indels** | **Coding indels** | **Minor allele frequency** |
| --- | --- | --- | --- | --- | --- | --- | --- | --- |
| AW | 5 | 99.20 | 16902379 | 15364944 | 130958 | 1537435 | 7837 | 0.265 |
| DM | 8 | 99.73 | 14934108 | 13665504 | 110928 | 1268604 | 5740 | 0.276 |
| GB | 85 | 99.67 | 15447595 | 14120129 | 115181 | 1327466 | 5936 | 0.277 |
| GD | 39 | 99.63 | 15296427 | 13972217 | 116960 | 1324210 | 6314 | 0.276 |
| GJ | 58 | 99.57 | 17850856 | 16210576 | 137681 | 1640280 | 8184 | 0.278 |
| GN | 52 | 99.68 | 15337289 | 14011138 | 115786 | 1326151 | 6179 | 0.278 |
| HB | 34 | 99.33 | 17489605 | 15898957 | 133806 | 1590648 | 7745 | 0.278 |
| JZ | 46 | 99.65 | 15035212 | 13726667 | 115126 | 1308545 | 6323 | 0.273 |
| LKZ | 10 | 99.52 | 16788986 | 15456348 | 140518 | 1332638 | 5956 | 0.271 |
| LZ | 9 | 99.75 | 15131647 | 13820796 | 111131 | 1310851 | 5802 | 0.274 |
| MX | 67 | 99.23 | 14864946 | 13573113 | 119384 | 1291833 | 6566 | 0.269 |
| QH | 44 | 99.66 | 15416367 | 14093769 | 122700 | 1322598 | 7001 | 0.275 |
| QK | 71 | 99.58 | 14831098 | 13587843 | 64958 | 1243255 | 3387 | 0.278 |
| QL | 44 | 99.44 | 14900961 | 13601805 | 114391 | 1299156 | 6103 | 0.269 |
| TJ | 64 | 99.51 | 17810295 | 16196615 | 136081 | 1613680 | 7906 | 0.280 |

**Supplementary Table S5.** Number of annotated SNPs in different gene regions

| **Population** | **Intergenic^a^** | **ncRNA^b^** | **UTR^c^** | **Intronic** | **Splice^d^** | **Exonic** | | |
| --- | --- | --- | --- | --- | --- | --- | --- | --- |
|  |  |  |  |  |  | **Non-synonymous^e^** | **Synonymous** | **Stop altering^f^** |
| AW | 9697376 | 50627 | 156374 | 5329609 | 11851 | 56380 | 61587 | 1140 |
| DM | 8633813 | 44230 | 138615 | 4737918 | 10216 | 47695 | 52031 | 986 |
| GB | 8909077 | 45404 | 143318 | 4907149 | 10675 | 49673 | 53748 | 1085 |
| GD | 8810356 | 45107 | 143395 | 4856399 | 10712 | 50606 | 54625 | 1017 |
| GJ | 10245895 | 52679 | 163899 | 5610422 | 12435 | 59611 | 64404 | 1231 |
| GN | 8829654 | 45840 | 143518 | 4876340 | 10644 | 49493 | 54601 | 1048 |
| HB | 10036758 | 51855 | 160573 | 5515965 | 12029 | 57738 | 62850 | 1189 |
| JZ | 8662814 | 45670 | 141314 | 4761743 | 10489 | 49908 | 53667 | 1062 |
| LKZ | 9704869 | 50250 | 163504 | 5397207 | 12448 | 61980 | 63820 | 2270 |
| LZ | 8725925 | 45060 | 140278 | 4798402 | 10172 | 47325 | 52636 | 998 |
| MX | 8541717 | 44845 | 141656 | 4725511 | 10669 | 51254 | 56424 | 1037 |
| QH | 8867500 | 46931 | 147083 | 4909555 | 11230 | 53001 | 57393 | 1076 |
| QK | 9545050 | 1212642 | 76213 | 2688980 | 5903 | 27911 | 30561 | 583 |
| QL | 8567773 | 44532 | 139510 | 4735599 | 10409 | 49530 | 53413 | 1039 |
| TJ | 10228358 | 52467 | 163195 | 5616514 | 12319 | 58694 | 63865 | 1203 |
| Mean | 9200462.333 | 125209.27 | 144163 | 4897820.9 | 10813.4 | 51386.6 | 55708.33333 | 1130.933333 |
| % | 63.50974441 | 0.8643053 | 0.9951408 | 33.8091 | 0.0746437 | 0.354715852 | 0.384548285 | 0.007806704 |

^a^ including "intergenic_region", "downstream_gene_variant", "upstream_gene_variant" given by snpEff

^b^ including "intragenic_variant", "non_coding_exon_variant"

^c^ including "3_prime_UTR_variant", "5_prime_UTR_premature_start_codon_gain_variant", "5_prime_UTR_variant"

^d^ including "splice_acceptor_variant", "splice_donor_variant", "splice_region_variant"

^e^ including "initiator_codon", "missense_variant"

^f^ including "stop_gained", "stop_lost", "stop_retained_variant"

**Supplementary Table S6.** Overlapped genes between altitude and geography by combining *F_st_* and *ZHp* in 15 sheep populations.

| **Numbers of genes** | **Chr.** | **Window_Start** | **Window_End** | **Fst** | **\|Zph\|** | **Genes within window** |
| --- | --- | --- | --- | --- | --- | --- |
| 8 | 13 | 32800001 | 32850000 | 0.0905701 | 1.7348 | ZEB1 |
|  | 13 | 49740001 | 49890000 | 0.0862544 | 1.7659 | LOC101110166 |
|  | 13 | 50400001 | 50550000 | 0.1209300 | 1.6659 | PANK2 |
|  | 13 | 50400001 | 50550000 | 0.1209300 | 1.6659 | RNF24 |
|  | 13 | 51550001 | 51700000 | 0.1029920 | 1.6957 | VPS16 |
|  | 13 | 51550001 | 51700000 | 0.0941852 | 1.6918 | PCED1A |
|  | 13 | 51560001 | 51710000 | 0.1037610 | 1.8468 | PTPRA |
|  | 13 | 62700001 | 62750000 | 0.1110400 | 1.8736 | RALY |
| 3 | 23 | 30760001 | 30910000 | 0.0820660 | 1.7687 | PSMA8 |
|  | 23 | 30770001 | 30920000 | 0.0618296 | 1.7615 | TAF4B |
|  | 23 | 60400001 | 60550000 | 0.1290100 | 1.5093 | LOC105605498 |
| 6 | 27 | 55450001 | 55600000 | 0.0889101 | 1.6948 | LOC101119700 |
|  | 27 | 56030001 | 56180000 | 0.1290100 | 1.5824 | LOC101119112 |
|  | 27 | 72870001 | 73020000 | 0.0889101 | 1.743 | APOOL |
|  | 27 | 72870001 | 73020000 | 0.1290100 | 1.7577 | ZNF711 |
|  | 27 | 72870001 | 73020000 | 0.0889101 | 1.7949 | HAG1(LOC101123097) |
|  | 27 | 72870001 | 73020000 | 0.1027100 | 1.8057 | POF1B |

**Supplementary Table S7.** Number of annotated INDEL in different gene regions

| **Population** | **Intergenic^a^** | **ncRNA^b^** | **UTR^c^** | **Intronic** | **Splice^d^** | **Exonic** | | |
| --- | --- | --- | --- | --- | --- | --- | --- | --- |
|  |  |  |  |  |  | **Frameshift** | **Nonframeshift^e^** | **Stop altering^f^** |
| AW | 955134 | 3657 | 19144 | 551663 | 2536 | 3561 | 1713 | 27 |
| DM | 786636 | 3051 | 15727 | 457450 | 2000 | 2449 | 1266 | 25 |
| GB | 821190 | 3156 | 16436 | 480748 | 2120 | 2431 | 1359 | 26 |
| GD | 819372 | 3168 | 16728 | 478628 | 2161 | 2716 | 1413 | 24 |
| GJ | 1019565 | 3784 | 19987 | 588760 | 2729 | 3644 | 1776 | 35 |
| GN | 819431 | 3158 | 16711 | 480672 | 2192 | 2554 | 1407 | 26 |
| HB | 987011 | 3773 | 19579 | 572540 | 2632 | 3424 | 1662 | 27 |
| JZ | 810239 | 3161 | 16477 | 472345 | 2132 | 2747 | 1415 | 29 |
| LKZ | 824188 | 3129 | 16352 | 483013 | 2148 | 2348 | 1423 | 37 |
| LZ | 812232 | 3119 | 16197 | 473501 | 1996 | 2414 | 1368 | 24 |
| MX | 798439 | 3120 | 16547 | 467161 | 2188 | 2904 | 1447 | 27 |
| QH | 815462 | 3284 | 17117 | 479734 | 2274 | 3147 | 1550 | 30 |
| QK | 859285 | 114089 | 8694 | 257800 | 1191 | 1390 | 797 | 9 |
| QL | 803850 | 3127 | 16355 | 469721 | 2118 | 2627 | 1333 | 25 |
| TJ | 1001592 | 3805 | 19708 | 580669 | 2653 | 3489 | 1730 | 34 |

^a^ including "intergenic_region", "downstream_gene_variant", "upstream_gene_variant" given by snpEff

^b^ including "intragenic_variant", "non_coding_exon_variant"

^c^ including "3_prime_UTR_variant", "5_prime_UTR_premature_start_codon_gain_variant", "5_prime_UTR_variant"

^d^ including "splice_acceptor_variant", "splice_donor_variant", "splice_region_variant".

^e^ including "disruptive_inframe_deletion", "disruptive_inframe_insertion", "inframe_deletion", "inframe_insertion"

^f^ including "stop_gained", "stop_lost"

**Supplementary Table S8.** Correlation between genes involving in the CSDE1-interation network

|  | **MAX** | **MYC** | **YBX1** | **HNRNPU** | **DHX9** | **PABPC1** | **PAIP1** | **STRAP** | **SYNCRIP** | **CSDE1** | **HAG1** |
| --- | --- | --- | --- | --- | --- | --- | --- | --- | --- | --- | --- |
| **MAX** | 1 | 0 | 0 | 0 | 0 | 0 | 0 | 0 | 0 | 0 | 0 |
| **MYC** | -0.1600372 | 1 | 0 | 0 | 0 | 0 | 0 | 0 | 0 | 0 | 0 |
| **YBX1** | 0.104814748 | -0.65618995 | 1 | 0 | 0 | 0 | 0 | 0 | 0 | 0 | 0 |
| **HNRNPU** | 0.811331952 | 0.15639305 | 0.12513544 | 1 | 0 | 0 | 0 | 0 | 0 | 0 | 0 |
| **DHX9** | 0.238095905 | -0.28248345 | 0.522449123 | 0.570741265 | 1 | 0 | 0 | 0 | 0 | 0 | 0 |
| **PABPC1** | 0.027644589 | 0.774844544 | -0.82439493 | -0.0535141 | -0.7419174 | 1 | 0 | 0 | 0 | 0 | 0 |
| **PAIP1** | -0.17802553 | 0.432819849 | -0.92283922 | -0.35474468 | -0.59318063 | 0.729431904 | 1 | 0 | 0 | 0 | 0 |
| **STRAP** | -0.78699378 | 0.055668249 | 0.409661901 | -0.43104476 | 0.219757778 | -0.38897935 | -0.38709091 | 1 | 0 | 0 | 0 |
| **SYNCRIP** | 0.60192023 | -0.45040611 | 0.758433857 | 0.706686965 | 0.723284988 | -0.65659264 | -0.8658138 | -0.05971316 | 1 | 0 | 0 |
| **CSDE1** | -0.42199686 | 0.919306076 | -0.73234285 | -0.22412566 | -0.51348692 | 0.817832075 | 0.63076961 | 0.155914382 | -0.74820606 | 1 | 0 |
| **HAG1** | -0.29452931 | 0.967907291 | -0.75952338 | -0.02224987 | -0.34476923 | 0.889264066 | 0.604425241 | 0.080198112 | -0.63401993 | 0.9714948 | 1 |
